# Sex differences in type of exercise associated with depression in South Korean adults

**DOI:** 10.1038/s41598-020-75389-8

**Published:** 2020-10-26

**Authors:** Hyunkyu Kim, Wonjeong Jeong, Junhyun Kwon, Youseok Kim, Sung-In Jang, Eun-Cheol Park

**Affiliations:** 1 Department of Preventive Medicine, Yonsei University College of Medicine, 50 Yonsei-ro, Seodaemun-gu, Seoul, 03722 Republic of Korea; 2 Institute of Health Services Research, Yonsei University, Seoul, Republic of Korea; 3 Department of Psychiatry, Yonsei University College of Medicine, Seoul, Republic of Korea; 4 Department of Public Health, Graduate School, Yonsei University, Seoul, Republic of Korea; 5 Department of Hospital Administration, Graduate School of Public Health, Yonsei University, Seoul, Republic of Korea

**Keywords:** Depression, Lifestyle modification, Preventive medicine

## Abstract

Exercise has been considered as treatment and a preventive modality to alleviate depressive symptoms, but sex differences regarding specific types of exercise in association with depression have not been clearly elucidated. Here, we investigated sex differences in the association between exercise type and depression in Korean adults. Data from the Korea National Health and Nutrition Examination Survey (KNHANES) were used for this study. A total of 13,914 participants who had filled in a Patient Health Questionnaire-9 (PHQ-9) were included. The subjects’ exercise status had been evaluated with questions on strength exercise and walking, and answers were analysed in the current study using multivariate logistic regression. Male participants who reported having done strength exercise more than once in a week were less likely to be depressed after adjusting for covariates assumed to affect depression levels [adjusted odds ratio (OR) 0.60, 95% CI 0.40–0.92]. In women, walking more than once during the previous week was associated with lower depression levels after covariate adjustments (adjusted OR 0.54, CI 0.34–0.87). This study identifies the relationship between exercise and the presence of depressive symptoms and finds sex differences in the types of exercise that correlate with depression in Korean adults.

## Introduction

Depression and, as a consequence, high suicide rates have become one of the big health problems and lead to a huge socioeconomic burden around the world^1–3^. South Korea also faces this problem, with its high suicide rate, and health policies have thus focused on managing depression and suicide for the last decades. Many studies have focused on biological and psychological causes and on a variety of treatment methods such as biological approaches, cognitive and behavioural therapy, and psychotherapy^4–7^. However, preventive methods for depression have not been widely established^8–11^. Among a variety of suggested strategies, exercise and physical activities have been hypothesized as one way of preventing depression^10^.

Reduced physical activity is one of the main features of depression and is also included in the diagnostic criteria^12^. Research has focused on reduced physical activity not only as a consequence of depressive symptoms but also as a potential target of modulating the disease. Previous studies have indeed suggested that exercise may play a role in the reduction of depressive symptoms in patients in specific target groups^13^. Furthermore, various exercise types have been evaluated for differences in their efficacy in alleviating depression. Most studies have focused on aerobic exercises, but a recent study suggested that anaerobic exercise also has the potential of reducing symptoms of depression^14^. These results provided the basis for many countries to include exercise in their depression guidelines, which has also happened in South Korea^15^.

In contrast to the obvious possibility of using exercise to treat depression, many aspects of the preventive ability of exercise remain unclear. Previous studies focused on initial physical activity and how it might lower the risk for depression^16^. However, sex differences or differences between exercise types have not been clearly identified, which makes it difficult to apply such findings in the clinical practice and in specific groups of patients. Previous studies showed sex difference in type of exercise that affect to depression treatment, suggesting the possibility of analogous association of exercise and depression prevalence in general population^17,18^. The association between types of exercise and depression therefore needs to be investigated, especially in groups of people with different demographic characteristics.

The aim of this study was to investigate the association between exercise types and depression in the Korean adult population, after adjusting for covariates assumed to affect depression levels. Furthermore, we also aimed to find sex differences in the relationship between exercise type and depression.

## Results

The general characteristics of the study population separated by sex are presented in Table 1. A total of 13,914 participants including 6010 men and 7904 women were included in the analysis. Of the participants, 2.96% of men and 6.73% of women fulfilled the depression criteria (i.e. a PHQ-9 score of 10 or higher). Participants who reported doing strength exercise more than once in a week showed a statistically lower depression prevalence than participants reporting no strength exercise. Also, participants who had walked more than 10 min at least once during the previous week had lower depression levels than participants who reported no walking. Educational attainment, equalized household income, marital status, smoking status, and stroke history were additionally identified as having statistically significant effects on depression.

**Table 1:** Socioeconomic and health-related characteristics of all study participants according to the presence/absence of depression.

Variables	Men (N = 6010)					Women (N = 7904)				
	Depressed (PHQ ≥ 10)		Not depressed (PHQ < 10)		p-value	Depressed (PHQ ≥ 10)		Not depressed (PHQ < 10)		p-value
	N	(%)	N	(%)		N	(%)	N	(%)	
**Strength exercise**					< 0.0001					0.0244
None	167	(4.3)	3709	(95.7)		454	(7.0)	6003	(93.0)	
≥ 1	47	(2.2)	2087	(97.8)		78	(5.4)	1369	(94.6)	
**Walking**					0.0125					0.0035
None	20	(6.01)	313	(93.99)		30	(11.11)	240	(88.89)	
≥ 1	193	(3.40)	5482	(96.60)		502	(6.58)	7132	(93.42)	
**Physical activity**					0.0990					0.5480
Low	90	(3.8)	2276	(96.2)		252	(6.9)	3388	(93.1)	
Moderate	97	(3.8)	2454	(96.2)		219	(6.4)	3205	(93.6)	
High	27	(2.5)	1066	(97.5)		61	(7.3)	779	(92.7)	
**Age**					0.415					< 0.0001
19–29	28	(3.4)	798	(96.6)		99	(9.5)	945	(90.5)	
30–39	45	(4.2)	1024	(95.8)		88	(6.4)	1288	(93.6)	
40–49	34	(3.1)	1066	(96.9)		55	(3.7)	1439	(96.3)	
50–59	31	(2.9)	1033	(97.1)		96	(6.0)	1496	(94.0)	
60–69	37	(3.5)	1017	(96.5)		105	(7.9)	1222	(92.1)	
≥ 70	39	(4.3)	858	(95.7)		89	(8.3)	982	(91.7)	
**Educational attainment**					< 0.0001					< 0.0001
Middle school and below	65	(5.6)	1090	(94.4)		227	(9.5)	2171	(90.5)	
High school	61	(3.7)	1572	(96.3)		136	(6.3)	2026	(93.7)	
University and above	88	(2.7)	3134	(97.3)		169	(5.1)	3175	(94.9)	
**Equalized household income**					< 0.0001					< 0.0001
Quartile 1 (low)	81	(8.9)	832	(91.1)		178	(12.9)	1205	(87.1)	
Quartile 2	57	(4.0)	1358	(96.0)		144	(7.3)	1828	(92.7)	
Quartile 3	42	(2.4)	1735	(97.6)		134	(6.1)	2080	(93.9)	
Quartile 4 (high)	34	(1.8)	1871	(98.2)		76	(3.3)	2259	(96.7)	
**Marital status**					< 0.0001					< 0.0001
Married	121	(2.8)	4253	(97.2)		268	(4.9)	5150	(95.1)	
Separated/divorced/widowed	34	(10.1)	304	(89.9)		151	(11.8)	1131	(88.2)	
Never married	59	(4.5)	1239	(95.5)		113	(9.4)	1091	(90.6)	
**Smoking status**					0.0006					< 0.0001
Non-smoker	30	(2.1)	1402	(97.9)		388	(5.5)	6642	(94.5)	
Smoker	184	(4.0)	4394	(96.0)		144	(16.7)	720	(83.3)	
**Alcohol use**					0.9297					0.7410
No	9	(3.5)	251	(96.5)		80	(7.0)	1070	(93.0)	
Yes	205	(3.6)	5545	(96.4)		452	(6.7)	6302	(93.3)	
**Residential area**					0.5647					0.5037
Urban	96	(3.4)	2716	(96.6)		249	(6.5)	3561	(93.5)	
Rural	118	(3.7)	3080	(96.3)		283	(6.9)	3811	(93.1)	
**BMI**					0.0002					0.0182
Underweight	15	(10.4)	129	(89.6)		40	(10.0)	359	(90.0)	
Normal weight	73	(3.8)	1837	(96.2)		232	(6.4)	3400	(93.6)	
Overweight	48	(3.1)	1490	(96.9)		101	(6.2)	1540	(93.8)	
Obesity	66	(3.1)	2030	(96.9)		125	(6.7)	1735	(93.3)	
Severe obesity	12	(3.7)	310	(96.3)		34	(9.1)	338	(90.9)	
**Stroke history**					< 0.0001					< 0.0001
No	200	(3.4)	5663	(96.6)		507	(6.5)	7271	(93.5)	
Yes	14	(9.5)	133	(90.5)		25	(19.8)	101	(80.2)	
**Participants**	177	(2.96)	5796	(97.04)		532	(6.73)	7372	(93.27)	

Variables are presented as numbers and percentages.

*PHQ-9* Patient health questionnaire-9, *BMI *body mass index, *Underweight *BMI < 18.5, *Normal weight *18.5 ≤ BMI < 23, *Overweight *23 ≤ BMI < 25, *Obesity *25 ≤ BMI < 30, *Severe obesity *30 ≤ BMI.

Table 2 shows the results of the multivariate logistic regression analysis on the association between depression and exercise. In men, participants who had done strength exercise more than once in a week were less likely to be depressed after adjusting for covariates [adjusted odds ratio (OR) 0.60, 95% CI 0.40–0.92]. Walking and physical activity were not statistically related with the prevalence of depression in men. High household income, married status, underweight, and no past history of stroke were found to correlate with lower depression prevalence in men, while age, educational attainment, alcohol use, residential area, overweight, and obesity were not related to depression risk in men. In women, walking at least once during the previous week showed an association with lower depression levels after adjustments for covariates (adjusted OR 0.54, CI 0.34–0.87). There were no association between strength exercise and prevalence of depression in women. Older age, higher educational attainment, and higher household income were inversely correlated with depression in women, while separated/divorced/widowed marital status, smoking, underweight, and stroke history were associated with a higher depression prevalence. Never married status, alcohol use, overweight, and obesity showed no statistical association with depression in women.

**Table 2:** Results of the multivariate logistic regression analysis on the association between exercise types and depression.

Variables	Men		Women	
	Depressed (PHQ ≥ 10)		Depressed (PHQ ≥ 10)	
	Adjusted OR	95% CI	Adjusted OR	95% CI
**Strength exercise**				
None	1.00		1.00	
≥ 1	0.60	(0.40–0.92)	0.84	(0.62–1.14)
**Walking**				
None	1.00		1.00	
≥ 1	0.55	(0.29–1.04)	0.54	(0.34–0.87)
**Physical activity**				
Low	1.00		1.00	
Moderate	1.13	(0.80–1.60)	1.04	(0.82–1.32)
High	1.04	(0.61–1.79)	1.36	(0.94–1.96)
**Age**				
19–29	1.00		1.00	
30–39	1.72	(0.92–3.19)	0.64	(0.39–1.05)
40–49	1.10	(0.55–2.19)	0.37	(0.21–0.65)
50–59	0.92	(0.38–2.24)	0.51	(0.28–0.90)
60–69	0.62	(0.27–1.43)	0.44	(0.24–0.81)
≥ 70	0.48	(0.19–1.20)	0.25	(0.13–0.48)
**Educational attainment**				
Middle school and below	1.00		1.00	
High school	0.83	(0.48–1.45)	0.63	(0.45–0.89)
University and above	0.68	(0.38–1.21)	0.43	(0.30–0.63)
**Equalized household income**				
Quartile 1 (low)	1.00		1.00	
Quartile 2	0.38	(0.23–0.61)	0.58	(0.43–0.78)
Quartile 3	0.23	(0.13–0.39)	0.60	(0.44–0.82)
Quartile 4 (high)	0.25	(0.14–0.43)	0.38	(0.26–0.54)
**Marital status**				
Married	1.00		1.00	
Separated/divorced/widowed	2.80	(1.73–4.53)	1.89	(1.45–2.47)
Never married	1.83	(1.02–3.28)	1.35	(0.85–2.15)
**Smoking status**				
Non-smoker	1.00		1.00	
Smoker	2.03	(1.21–3.39)	2.89	(2.24–3.73)
**Alcohol use**				
No	1.00		1.00	
Yes	1.16	(0.53–2.55)	0.92	(0.67–1.27)
**Residential area**				
Urban	1.00		1.00	
Rural	0.95	(0.68–1.32)	1.01	(0.80–1.27)
**BMI**				
Underweight	2.56	(1.24–5.29)	1.63	(1.05–2.53)
Normal weight	1.00		1.00	
Overweight	1.11	(0.69–1.78)	0.81	(0.59–1.12)
Obesity	1.10	(0.71–1.70)	0.91	(0.68–1.21)
Severe obesity	1.44	(0.72–2.87)	1.22	(0.77–1.95)
**Stroke history**				
No	1.00		1.00	
Yes	2.54	(1.24–5.20)	3.78	(2.00–7.12)

*PHQ-9* patient health questionnaire-9, *BMI *body mass index, *Underweight *BMI < 18.5, *Normal weight *18.5 ≤ BMI < 23, *Overweight *23 ≤ BMI < 25, *Obesity *25 ≤ BMI < 30, *Severe obesity *30 ≤ BMI, *OR* odds ratio, *CI *confidence interval.

The subgroup analyses were conducted to assess the combined effects of strength exercise and other sociodemographic variables on depression, as shown in Table 3. In married men, strength exercise was statistically associated with a lower depression prevalence (adjusted OR 0.48, CI 0.27–0.84), but no relationship was found for other marital statuses. Table 4 shows the results from the subgroup analyses on the combined effects of walking during the past week and sociodemographic variables on the prevalence of depression. In women, walking was found to be related to depression in the youngest (19–29) and the oldest (≥ 70) age group but not in other groups. Walking also showed an association with lower prevalences of depression in subgroups using alcohol and smoking, compared to non-smokers and non-drinkers.

**Table 3:** Subgroup analysis of the association between strength exercise and depression stratified by sociodemographic variables.

Variables	Men			Women		
	No strength exercise	Strength exercise ≥ 1		No strength exercise	Strength exercise ≥ 1	
	Adjusted OR	Adjusted OR	95% CI	Adjusted OR	Adjusted OR	95% CI
**Physical activity**						
Low	1.00	0.86	(0.43–1.69)	1.00	0.66	(0.35–1.23)
Moderate	1.00	0.53	(0.29–0.96)	1.00	0.85	(0.54–1.33)
High	1.00	0.53	(0.22–1.27)	1.00	1.17	(0.64–21.35)
**Age**						
19–29	1.00	1.13	(0.41–3.13)	1.00	1.47	(0.80–2.70)
30–39	1.00	0.44	(0.20–1.00)	1.00	0.67	(0.33–1.36)
40–49	1.00	0.63	(0.28–1.43)	1.00	0.53	(0.19–1.46)
50–59	1.00	0.46	(0.17–1.27)	1.00	0.76	(0.33–1.72)
60–69	1.00	0.37	(0.12–1.17)	1.00	0.63	(0.33–1.22)
≥ 70	1.00	0.56	(0.22–1.42)	1.00	0.32	(0.12–0.81)
**Educational attainment**						
Middle school and below	1.00	0.93	(0.44–1.96)	1.00	0.70	(0.39–1.27)
High school	1.00	0.57	(0.26–1.25)	1.00	0.84	(0.48–1.46)
University and above	1.00	0.54	(0.31–0.95)	1.00	0.94	(0.58–1.54)
**Equalized household income**						
Quartile 1 (low)	1.00	0.94	(0.48–1.84)	1.00	1.10	(0.54–2.22)
Quartile 2	1.00	0.40	(0.17–0.95)	1.00	0.68	(0.41–1.13)
Quartile 3	1.00	0.37	(0.16–0.8)	1.00	0.78	(0.43–1.42)
Quartile 4 (high)	1.00	0.66	(0.25–1.76)	1.00	0.94	(0.48–1.84)
**Marital status**						
Married	1.00	0.48	(0.27–0.84)	1.00	0.73	(0.46–1.18)
Separated/divorced/widowed	1.00	0.59	(0.20–1.73)	1.00	0.67	(0.38–1.19)
Never married	1.00	0.77	(0.36–1.63)	1.00	1.14	(0.63–2.06)
**Smoking status**						
Non-smoker	1.00	1.01	(0.40–2.54)	1.00	0.88	(0.61–0.28)
Smoker	1.00	0.52	(0.33–0.81)	1.00	0.80	(0.47–1.37)
**Alcohol use**						
No	1.00	0.78	(0.08–8.09)	1.00	0.64	(0.27–1.51)
Yes	1.00	0.61	(0.40–0.94)	1.00	0.87	(0.63–1.20)
**Residential area**						
Urban	1.00	0.47	(0.24–0.93)	1.00	0.78	(0.50–1.24)
Rural	1.00	0.76	(0.34–1.28)	1.00	0.90	(0.60–1.36)
**BMI**						
Underweight	1.00	1.44	(0.16–12.68)	1.00	0.86	(0.23–3.22)
Normal weight	1.00	0.49	(0.24–0.998)	1.00	0.89	(0.48–1.362)
Overweight	1.00	0.68	(0.32–1.46)	1.00	0.41	(0.15–1.10)
Obesity	1.00	0.39	(0.17–0.92)	1.00	1.11	(0.59–2.09)
Severe obesity	1.00	1.94	(0.24–15.82)	1.00	0.24	(0.05–1.07)
**Stroke history**						
No	1.00	0.58	(0.38–0.90)	1.00	0.88	(0.65–1.20)
Yes	1.00	0.68	(0.01–38.75)	1.00	0.25	(0.02–2.92)

*PHQ-9* patient health questionnaire-9, *BMI *body mass index, *Underweight *BMI < 18.5, *Normal weight *18.5 ≤ BMI < 23, *Overweight *23 ≤ BMI < 25, *Obesity *25 ≤ BMI < 30, *Severe obesity *30 ≤ BMI, *OR *odds ratio, *CI* confidence interval.

**Table 4:** Subgroup analysis of the association between walking and depression stratified by sociodemographic variables.

Variables	Men			Women		
	No walking	Walking ≥ 1		No walking	Walking ≥ 1	
	Adjusted OR	Adjusted OR	95% CI	Adjusted OR	Adjusted OR	95% CI
**Physical activity**						
Low	1.00	0.49	(0.21–1.18)	1.00	0.59	(0.33–1.06)
Moderate	1.00	1.54	(0.51–4.68)	1.00	0.50	(0.21–1.17)
High	1.00	0.20	(0.05–0.75)	1.00	0.37	(0.05–2.70)
**Age**						
19–29	1.00	0.90	(0.12–6.86)	1.00	0.27	(0.12–0.63)
30–39	1.00	0.29	(0.11–0.78)	1.00	0.64	(0.17–2.34)
40–49	1.00	1.05	(0.35–3.20)	1.00	0.93	(0.18–4.84)
50–59	1.00	3.34	(0.16–69.82)	1.00	0.53	(0.17–1.60)
60–69	1.00	0.56	(0.10–3.17)	1.00	0.41	(0.15–1.09)
≥ 70	1.00	0.23	(0.08–0.71)	1.00	0.27	(0.18–0.63)
**Educational attainment**						
Middle school and below	1.00	0.42	(0.13–1.36)	1.00	0.35	(0.18–0.65)
High school	1.00	0.58	(0.25–1.34)	1.00	0.70	(0.26–1.90)
University and above	1.00	0.55	(0.20–1.51 )	1.00	0.66	(0.24–1.81)
**Equalized household income**						
Quartile 1 (low)	1.00	0.45	(0.11–1.86)	1.00	0.41	(0.19–0.86)
Quartile 2	1.00	0.70	(0.24–1.98)	1.00	0.38	(0.15–0.95)
Quartile 3	1.00	0.31	(0.12–0.83)	1.00	0.45	(0.17–1.20)
Quartile 4 (high)	1.00	0.62	(0.14–2.76)	1.00	2.61	(0.59–11.48)
**Marital status**						
Married	1.00	0.65	(0.30–1.43)	1.00	0.59	(0.33–1.08)
Separated/divorced/widowed	1.00	0.12	(0.02–0.57)	1.00	0.59	(0.21–1.70)
Never married	1.00	0.64	(0.13–3.26)	1.00	0.48	(0.11–2.23)
**Smoking status**						
Non-smoker	1.00	0.36	(0.07–1.92)	1.00	0.69	(0.40–1.19)
Smoker	1.00	0.59	(0.29–1.20)	1.00	0.28	(0.11–0.73)
**Alcohol use**						
No	1.00	0.08	(0.01–0.44)	1.00	0.37	(0.13–1.12)
Yes	1.00	0.59	(0.30–1.15)	1.00	0.56	(0.32–0.95)
**Residential area**						
Urban	1.00	1.40	(0.42–4.68)	1.00	0.50	(0.22–1.13)
Rural	1.00	0.39	(0.17–0.86)	1.00	0.60	(0.33–1.08)
**BMI**						
Underweight	1.00	*	(*–*)	1.00	1.28	(0.12–13.11)
Normal weight	1.00	0.50	(0.22–1.14)	1.00	0.72	(0.32–1.59)
Overweight	1.00	0.52	(0.08–3.24)	1.00	0.22	(0.10–0.49)
Obesity	1.00	0.71	(0.22–2.25)	1.00	0.52	(0.20–1.35)
Severe obesity	1.00	0.21	(0.01–5.61)	1.00	0.33	(0.04–2.56)
**Stroke history**						
No	1.00	0.20	(0.02–2.51)	1.00	0.53	(0.33–0.87)
Yes	1.00	0.54	(0.28–1.04)	1.00	0.16	(0.01–2.35)

*PHQ-9 *patient health questionnaire-9, *BMI *body mass index, *Underweight *BMI < 18.5, *Normal weight *18.5 ≤ BMI < 23, *Overweight *23 ≤ BMI < 25, *Obesity* 25 ≤ BMI < 30, *Severe obesity *30 ≤ BMI, *OR *odds ratio, *CI *confidence interval.

*Due to the sparsity of the data, OR was > 999,999 which does not represent a proper value.

Table 5 shows the results from subgroup analyses among somatic symptom items of PHQ-9 and exercise. Walking was associated with sleep disturbance (item 3), low energy level (item 4), poor appetite (item 5) in men and low energy level, agitation/retardation (item 8) in women. Strength exercise was only associated with low energy level in men. The results of subgroup analyses among exercise and cognitive/affective symptoms were shown in Table 6. Poor concentration (item 7) was not associated with any type of exercise. Other cognitive/affective items except item 7 were associated with walking in women. Item 1 (little interest or pleasure in doing things) was associated with strength exercise in men. Suicidal or self-mutilating ideation (item 9) was associated with walking in both sexes.

**Table 5:** Dependent subgroup analysis of the association among strength exercise , walking and somatic symptoms items of PHQ-9.

Variables		PHQ-9 Item-3 ≥ 1		PHQ-9 Item-4 ≥ 1		PHQ-9 Item-5 ≥ 1		PHQ-9 Item-8 ≥ 1	
		Adjusted OR	95% CI	Adjusted OR	95% CI	Adjusted OR	95% CI	Adjusted OR	95% CI
Men	**Strength exercise**								
	None	1.00		1.00		1.00		1.00	
	≥ 1	1.07	(0.83–1.37)	0.71	(0.55–0.92)	0.73	(0.50–1.07)	0.50	(0.24–1.03)
	**Walking**								
	None	1.00		1.00		1.00		1.00	
	≥ 1	0.53	(0.34–0.82)	0.47	(0.31–0.73)	0.43	(0.24–0.76)	0.67	(0.28–1.64)
Women	**Strength exercise**								
	None	1.00		1.00		1.00		1.00	
	≥ 1	1.00	(0.81–1.23)	0.87	(0.71–1.07)	0.82	(0.61–1.09)	0.59	(0.29–1.18)
	**Walking**								
	None	1.00		1.00		1.00		1.00	
	≥ 1	1.21	(0.81–1.82)	0.65	(0.46–0.93)	0.78	(0.49–1.25)	0.23	(0.11–0.50)

*PHQ-9* patient health questionnaire-9, *OR *odds ratio, *CI *confidence interval.

**Table 6:** Dependent subgroup analysis of the association among strength exercise , walking and cognitive/affective items of PHQ-9.

Variables		PHQ-9 Item-1 ≥ 1		PHQ-9 Item-2 ≥ 1		PHQ-9 Item-6 ≥ 1		PHQ-9 Item-7 ≥ 1		PHQ-9 Item-9 ≥ 1	
		Adjusted OR	95% CI	Adjusted OR	95% CI	Adjusted OR	95% CI	Adjusted OR	95% CI	Adjusted OR	95% CI
Men	**Strength exercise**										
	None	1.00		1.00		1.00		1.00		1.00	
	≥ 1	0.55	(0.40–0.76)	0.77	(0.50–1.17)	0.71	(0.45–1.12)	0.58	(0.31–1.08)	0.73	(0.27–1.98)
	**Walking**										
	None	1.00		1.00		1.00		1.00		1.00	
	≥ 1	0.69	(0.39–1.23)	0.88	(0.41–1.88)	0.59	(0.31–1.13)	0.59	(0.26–1.32)	0.23	(0.07–0.83)
Women	**Strength exercise**										
	None	1.00		1.00		1.00		1.00		1.00	
	≥ 1	1.05	(0.79–1.38)	0.94	(0.67–1.33)	0.62	(0.41–0.95)	0.80	(0.48–1.34)	0.61	(0.34–1.10)
	**Walking**										
	None	1.00		1.00		1.00		1.00		1.00	
	≥ 1	0.50	(0.32–0.79)	0.50	(0.30–0.84)	0.54	(0.32–0.90)	0.70	(0.37–1.33)	0.44	(0.23–0.86)

*PHQ-9* patient health questionnaire-9, *OR *odds ratio, *CI *confidence interval.

## Discussion

In the present study, we identified that current depression levels are associated with exercise during the previous week in Korean adults. Furthermore, we found a sex difference in exercise types that correlated with the prevalence of depression. In men, participants who reported doing strength exercise once or more a week showed a lower prevalence of depression. In contrast, walking was associated with a lower prevalence of depression in women.

Previous studies showed associations between high physical activity and low prevalences of depression that are generally in agreement with our results. Harvey et al. reported a relationship between initial exercise and depression and anxiety after 11 years of observation in a cohort study of 33,908 healthy adults in Norway^16^. Baseline physical activity reduced depression levels by 12% in that study. One cohort study conducted in South Korea in a sample of 107,901 participants suggested that 1200–3000 METs/week physical activity were associated with a lower risk of depression^19^. The results from a systematic review showing that depressive symptoms increased significantly when exercise was stopped also suggest a preventive effect of exercise on depression^20^. The results of these previous studies indicate that the correlation found in the current study is not only the result of the patients’ symptoms of depression but can be explained by the impact of exercise on the prevalence of depression.

How exercise may prevent depression has not yet been clearly established. However, previous studies have proposed several hypotheses. Depression is known to be related to atrophy of brain structures including the hippocampus, anterior cingulate cortex, and prefrontal cortex^21^. Some studies suggested that exercise increases the volume of the hippocampus, anterior accumbens, and prefrontal cortex, thus leading to anti-depressive effects^22–24^. Exercise has also been found to increase brain-derived neurotrophic factor (BDNF) levels in previous studies. BDNF has an effect on regeneration and increases hippocampus functions, and a decrease in BDNF has been found to be related to depression in the elderly, suggesting that exercise may be able to reverse and prevent depressive symptoms by increasing BDNF^25^. Elevated serotonin levels after exercise have also been suggested as one possible mechanism of anti-depressive effects. Activated muscles require branched-chain amino acids as their substrates, and relative high concentrations of a single amino acid such as tryptophan in the serum may lead to high concentrations in the cerebrospinal fluid, resulting in increased levels of serotonin and dopamine which can reduce depression^26,27^.

This study shows not only that exercise is effective, but that different types of exercise are effective in men and women. In men, strength exercise was associated with reduced depression, while in women, walking was significantly correlated with the prevalence of depression. The number of studies that show differences in the types of exercise that have an effect on depression according to sex is limited, but Balkin et al. reported that in women, only aerobic, but not anaerobic, exercise alleviated depression^18^. In one study conducted in obese adolescent women, only aerobic exercise was effective in improving depressive symptoms, while anaerobic and leisure exercise were not effective^28^. Previous studies thus suggest that aerobic exercise is superior to anaerobic exercise in reducing depressive symptoms, which is similar to our results. Kianian et al. reported that in men, all kinds of exercise show reducing effects on depressive symptoms, but that anaerobic exercise showed a larger effect than aerobic exercise^29^. One possible explanation for this difference is that decreased muscle mass is significantly related to depressive symptoms, but only in men, which may explain the difference in the effect of anaerobic exercise between men and women^30^.

From the subgroup analyses stratified by age, the results showed that walking was associated with low depression prevalence in both sex over 70 years old and strength exercise was associated with low depression in women over 70 years old. One previous meta-analysis showed that exercise has beneficial effect on treating depression in older adults^31^. Physical activities and muscle mass would be reduced in old people, thus exercise could reduce the depressive symptoms more in older people than younger population.

This study has several limitations. First, the use of cross-sectional data does not allow us to draw clear cause-effect conclusions regarding exercise and the prevalence of depression. Second, data were collected via a survey, and the results might have been affected by a recall bias. Third, a number of other important confounding variables may not have been considered. For example, a family history of mental disorder, medication history, or comorbid psychiatric disorders might affect the prevalence of depression, but as these data were not included in the KNHANES, we could not include such variables into our analysis. Fourth, the KNHANES is a sample survey of the general population in South Korea, and people who were in hospitals or facilities for severe symptoms of depression at the time of the study were not included. It is possible that patients with severe depression would show different effects of exercise.

Despite these limitations, this study provides additional evidence that there is sex difference in type of exercise which is related to the depression prevalence. This result suggests that further studies or health policies on exercise for treating/preventing depression should consider the sex difference of the type of exercise. In addition, the association between exercise and depression was more significant in ages over 70 which suggest that exercise could be considered as a useful method to modulate and prevent the depression in older population.

In conclusion, this study identified the relationship between exercise and the presence of depression in Korean adult individuals and, in particular, a sex differences in the type of exercise that correlated with depression in men and women, with strength exercise in men and walking in woman correlating with lower depression rates. Further research using prospective designs that allow for a clear assessment of sex differences in exercise types potentially effective in the prevention of depression should follow to validate these findings.

## Methods

### Study population and data

The data analysed in this study was taken from the 2014–2018 Korea National Health and Nutrition Examination Survey (KNHANES). The KNHANES is a nationwide survey on health and nutrition conducted by the Korea Centers for Disease Control and Prevention. The purpose of the KNHANES is to evaluate the health status, health behaviour, and nutritional status of people in South Korea in order to provide basic data for developing health policies, such as the setting and evaluation of goals of comprehensive plans developed by the Korea Health Promotion Institute as well as other health promotion programs. The survey is conducted annually, but items change slightly every year. Questionnaires on depressive symptoms are included every even year, and we therefore included the data from 2014, 2016, and 2018 in our analyses. All participants signed an informed consent when they participated in the KNHANES. The survey complies with the Declaration of Helsinki, and was exempted from institutional review board approval, by government regulation. As the KNHANES provides de-identified and publicly accessible data, ethical approval was not required for the current study.

### Measures

#### Patient Health Questionnaire-9 (PHQ-9)

The Patient Health Questionnaire-9 is a self-report questionnaire for the screening for depression, and includes nine questions that are components of the Primary Care Evaluation of Mental Disorders instrument^32^. The PHQ-9 has been established as a reliable and valid screening tool for depression and for evaluating disease severity^33^. The PHQ-9 is popular and widely used because of its briefness, although it has the limitations of unstructured diagnostic tools. Each question is scored from 0 to 3, resulting in a total score from 0 to 27. A score of 10 has been suggested as a valid cut-off for detecting major depression in the initial study, which yielded a sensitivity of 88% and a specificity of 88%^33^. The Korean version of the PHQ-9 was standardized by An et al., and has shown an internal consistency and test–retest reliability of 0.95 and 0.91, respectively^34^. The Sensitivity and specificity of the Korean PHQ-9 with a cut-off level of 10 were 87.8% and 97.4%^34^. In this study, we therefore defined depression as a PHQ-9 level of 10 or higher.

#### Exercise variables

Participants answered questions on whether they had done any kind of strength exercises or had walked for more than 10 min during the recent weeks. The question about strength exercises asked whether the subjects have done any kind of the exercise of muscle including push-ups, sit-ups, weight lifting, pull ups and etc. Each participant was classified as one who exercised more than once a week, and one who did not exercise more than once. To adjust for the total physical activity of the patients, the International Physical Activity Questionnaire (IPAQ) was also administered, which asked for recent occupational and leisure-time physical activity. Overall physical activity was divided into three categories using metabolic equivalent task (MET) units, as has been suggested by suggested scoring protocol and in previous studies^35–37^.

#### Covariates

Age, educational attainment, equalized household income, marital status, and residential area were included in the demographic and socioeconomic covariates. Health-related covariates including smoking status, alcohol use, and stroke history were also used to adjust the analysis. Stroke history was included in the health-related covariates, because it is related to physical restriction as well as to depression occurrence^38^.

### Statistical analysis

Chi-square tests were used to investigate and compare the general characteristics of the study population. A multivariate logistic regression analysis was conducted to examine the relationship between performed exercise and depression. Potential compound variables including demographic, socio-economic, and health-related characteristics were included in the analysis. Subgroup analyses were performed to investigate the combined effects of performed exercise and other covariates on depression. The results are presented as odds ratios (ORs) and confidence intervals (CI) to compare prevalences of depression between groups with different exercise statuses. The analyses were performed with stratified sampling variables (kstrata) and weighted variables suggested by the KNHANES. All analyses were carried out using SAS software version 9.4 (SAS Institute, Cary, North Carolina, USA), and results were considered as statistically significant at a p-value < 0.05.

## Data Availability

The data analysed in this study were taken from the 2014–2018 KNHANES which is available to the public. All data can be downloaded from the KNHANES official website (https://knhanes.cdc.go.kr/).
